# Cross-cultural adaptation and validation of the Pelvic Floor Impact Questionnaire (PFIQ-7) short form in Urdu: a comprehensive linguistic and psychometric analysis

**DOI:** 10.1186/s41687-025-00932-y

**Published:** 2025-09-29

**Authors:** Anum Malik, Urooj Kashif, Nazish Baloch, Amir Raza, Novera Chughtai

**Affiliations:** 1 Fellow Urogynecology and Pelvic Reconstructive Surgery, AGA Khan University, Karachi, Pakistan; 2 AGA Khan University, Stadium Road, PO. Box. 3500, Karachi, 74800 Pakistan

**Keywords:** Pelvic floor disorders (PFDs), Quality of life, Patient-reported outcomes measures (PROMs), Urinary incontinence (UI), Pelvic organ prolapse (POP)

## Abstract

**Background:**

Pelvic floor disorders (PFDs) affect women’s physical, psychological, and social well-being, particularly in low- and middle-income countries. The PFIQ-7, a key patient-reported outcome (PROM) measure, assesses symptom severity and quality of life but lacks an Urdu adaptation. This study aims to translate, culturally adapt, and validate the PFIQ-7 for Urdu-speaking women to improve its clinical and research utility.

**Methodology:**

This cross-sectional, observational study was conducted in two phases: Phase 1 focused on translating and culturally adapting the PFIQ-7, while Phase 2 assessed its psychometric properties. A total of 108 Urdu-literate women participated. The PFIQ-7 underwent rigorous translation following international guidelines, including forward and backward translation, consensus meetings, and pilot testing. The study evaluated reliability (Cronbach’s alpha, interclass correlation) and validity (content, criterion, and construct validity) through expert review, S-POP-Q correlation, and known-group comparisons.

**Results:**

The Urdu PFIQ-7 demonstrated excellent internal consistency (Cronbach’s alpha = 0.884) and test-retest reliability (ICC = 0.995). Strong criterion validity was evidenced by significant correlations between PFIQ-7 scores and S-POP-Q findings (SCC = 0.67; *p* < 0.005). Construct validity was confirmed, as patients with higher S-POP-Q stages and urinary incontinence reported significantly higher PFIQ-7 scores compared to asymptomatic participants (*p* < 0.0005). Pilot testing confirmed the questionnaire’s clarity, cultural relevance, and feasibility, with a completion time of approximately four to five minutes.

**Conclusions:**

The Urdu PFIQ-7 is a reliable and valid tool for assessing PFD’s impact on quality of life, supporting its use in clinical and research settings in Pakistan. Future research should explore its utility in rural and community-based settings to enhance generalizability.

## Abstract (plain language summary)

Pelvic floor problems, like urinary incontinence and organ prolapse, affect millions of women and can significantly reduce their quality of life—especially in countries like Pakistan, where access to care may be limited. To better understand how these issues affect women’s daily lives, doctors and researchers use specially designed surveys that let patients describe their own experiences. One such survey, the Pelvic Floor Impact Questionnaire (PFIQ-7), asks about how these problems interfere with things like physical activity, emotions, and social life. However, this tool wasn’t available in Urdu, the main language spoken in Pakistan. This study aimed to translate and adapt the PFIQ-7 into Urdu for better use with Urdu-speaking women. The questionnaire was carefully translated and then tested with over 100 women at a hospital in Karachi. This helped ensure that the questions were easy to understand and provided reliable results. Our findings showed that the Urdu version of the PFIQ-7 is clear, easy to understand, and accurately measures how pelvic floor issues affect quality of life. This means it can now be used by doctors and researchers in Pakistan to better understand and support women with these health issues. It also lays the foundation for broader public health studies in other Urdu-speaking communities.

## Introduction

Pelvic floor disorders (PFDs) are chronic health ailments impacting women and encompass pelvic organ prolapse (POP), urinary incontinence (UI), and fecal incontinence (FI). These disorders are a significant global health concern [1], with a notable prevalence in low- and middle-income countries, where approximately 25% of women experience one or more PFDs [2]. In a population-centered study from rural areas of Pakistan, the prevalence of POP among symptomatic females aged 15 years or above, confirmed through clinical examination, was reported to be 10.3% [3].

PFDs have far-reaching effects on women’s physical, sexual, social, and psychological well-being, ultimately diminishing their health-related quality of life (HRQoL) [1, 2, 4, 5]. Given the substantial health and economic burden of these conditions, effective management strategies are critical. Evaluating the intensity of symptoms and their impact on HRQoL is essential for guiding treatment and monitoring progress in women with PFDs [6, 7]. In the field of urogynecology, the use of patient-reported outcome measures (PROMs) has become a cornerstone for assessing these parameters [8].

PROMs are patient-centered tools that capture health outcomes directly reported by patients without any interpretation or alteration by clinicians [9]. In the context of PFDs, numerous PROMs have been designed to assess symptom load, functional state, and HRQoL [10]. These tools are gaining prominence as they provide insights into treatment efficacy and patient satisfaction. Specifically, evaluating both the intensity of symptoms and the multifaceted effects of PFDs on women’s lives is crucial for determining the efficiency of non-surgical and surgical interventions [11].

Among the available PROMs, the Pelvic Floor Impact Questionnaire (PFIQ-7) is a widely used, psychometrically robust, and reliable tool for measuring the impact of PFDs on HRQoL [12]. Created in 2005 by Barber et al. [12], the PFIQ-7 includes three subscales: the Urinary Impact Questionnaire (UIQ), the Colo-Rectal-Anal Impact Questionnaire (CRAIQ), and the Pelvic Organ Prolapse Impact Questionnaire (POPIQ), which evaluate four important facets of a woman’s life: travel, social interactions, physical activity, and emotional well-being. The PFIQ-7 has been translated and validated in several languages due to its clinical and scientific significance [13–25]. However, to date, the PFIQ-7 in Urdu has not been validated, limiting its utility. To effectively use PROM in a new language, rigorous translation, cultural adaptation, and validation are essential to ensure accuracy and reduce bias [26]. Validating the PFIQ-7 in Urdu will enable accurate symptom reporting, support research, and improve care for Urdu-speaking women who have pelvic floor disorders. Thus, our objective was to translate, culturally adapt the PFIQ-7 into Urdu and assess its measurement properties, including reliability and validity.

## Materials and methods

This research was carried out in Karachi at the Hospital’s Obstetrics & Gynaecology Department, following approval from the Ethical Review Committee (2024-9274-28022). The cross-sectional, observational study was conducted in two phases. Phase 1 involved the translation and cultural adaptation of the PFIQ-7 instrument into Urdu, while Phase 2 focused on evaluating its psychometric properties.

### Measurement instruments

The PFIQ-7 questionnaire consists of 21 items that assess the effects of PFDs on various facets of life, including household chores, physical activities, social participation, emotional well-being, travel, entertainment, and frustration. A scale from 0 (“not at all”) to 3 (“quite a bit”) was used to score the responses. The final score (range 0–300) was derived by summing the scale scores, with higher values suggesting a stronger impact on daily life.

### Phase 1: Translation and cultural adaptation

The linguistic validation process adhered to established guidelines to ensure conceptual and technical equivalence between the original and target language [27]. The following steps were performed:

#### Forward translation

Two independent translators, fluent in both English and Urdu, translated the PFIQ-7 into Urdu.

#### Consensus meeting

The study team and translators convened a consensus meeting to compare the translations and develop the initial Urdu version.

#### Back translation

Two more translators proficient in both languages backtranslated the original Urdu version into English.

#### Revision

A second consensus meeting was conducted with the translators and research team to compare the back-translation with the original PFIQ-7 and revise the Urdu version accordingly.

#### Pilot testing

The revised Urdu version was tested on 10 women with PFDs to ensure clarity and relevance. Any challenges in understanding the questions or response options were recorded and addressed. The final Urdu version of the PFIQ-7 was then finalized.

### Phase 2: Psychometric evaluation

Women with and without pelvic floor disorders (PFDs) were recruited from gynecology outpatient clinics. Eligible participants were Urdu-literate women aged 18 or older who had not undergone pelvic or gynecological surgery in the past six months. Women were excluded if they had a diagnosed psychiatric or cognitive disorder, were unable to speak, read, write, or understand Urdu, or had a history of pelvic or anti-incontinence surgery within the last six months. Additional exclusion criteria included current pregnancy, being less than six weeks post-partum, being under 18 years of age, or submitting an incomplete questionnaire. The sample size was calculated as per the recommendations of the Consensus-based Standards for the Selection of Health Measurement Instruments (COSMIN) [28], which suggests including at least five participants per questionnaire item. As the PFIQ-7 has 21 items, a minimum sample size of 105 participants was required. The final sample size was 116 participants to accommodate a 10% non-response rate. Demographic and clinical details, including age, parity, UI, FI, and simplified Pelvic Organ Prolapse Quantification (S-POPQ) stages, were recorded. Informed consent was obtained, and an Urdu version of the consent form was provided.

Participants completed the PFIQ-7 questionnaire in a private room during their initial visit. After completing the questionnaire, all participants underwent a gynaecological examination conducted by blinded team members. The examination included the cough stress test and S-POPQ staging, which assessed the degree of vaginal wall descent while coughing or Valsalva.

The S-POPQ stages were defined as follows:

#### Stage 0:

No prolapse.

#### Stage 1:

The leading point remained at least 1 cm above the hymenal ring.

#### Stage 2:

The leading point descended to within 1 cm above or below the hymenal ring.

#### Stage 3:

Outside the hymenal ring, the leading point descended more than 1 centimeter without complete vaginal wall eversion.

#### Stage 4:

Complete vaginal vault eversion or procidentia uteri.

To assess test-retest reliability, women who had PFDs were requested to fill out the PFIQ-7 again after two weeks, provided no symptomatic changes or interventions had occurred. This interval was chosen to minimize recall bias while ensuring symptom stability. The follow-up assessment was conducted using a standardized version of the PFIQ-7, administered by team members.

### Statistical analysis

Data were analyzed using the Statistical Package for the Social Sciences (SPSS), version 19.The psychometric properties of the PFIQ-7 questionnaire were evaluated through methodological testing, focusing on reliability and validity. Internal consistency was measured using Cronbach’s alpha, with values above 0.7 considered acceptable. Additionally, test-retest reliability was analyzed over two weeks using intraclass correlation coefficients (ICC), where values exceeding 0.70 indicated satisfactory reliability.

An expert panel assessed content validity to determine how well the questionnaire accurately represented the intended construct. Criterion validity was examined by measuring the correlation between PFIQ-7 scores and S-POPQ scores using Spearman’s correlation coefficient (SCC), assessing the questionnaire’s alignment with an established gold standard, with the hypothesis that higher S-POPQ scores would correlate with poorer HRQoL.

Hypothesis evaluation and known-group validity were used to evaluate construct validity. To assess known-group validity, participants were categorized based on the presence or absence of specific pelvic floor conditions. These groups included: women with and without POP, identified using a S-POPQ score greater than 1, women with and without UI, and women with and without FI. A hypothesis was made that PFIQ-7 scores would correlate with S-POPQ scores and that patients with higher S-POPQ scores would report poorer quality of life, as reflected by higher PFIQ-7 scores. Additionally, it was expected that the POPIQ-7 scores of POP patients would be higher than those without POP patients. Group differences in PFIQ-7 total and subscale scores were analyzed using the Mann-Whitney U test. Construct validity was deemed satisfactory if at least 75% of the predefined hypotheses were supported.

## Results

### Characteristics of participants

A total of 116 participants represented the planned recruitment target, while the final sample of 108 reflected the actual number of complete and analyzable responses. Table 1 summarizes the demographic and clinical characteristics of the study participants. The mean age was 54.69 ± 14.79 years (range: 27–85). Most participants had a BMI in the 25.1–29.9 kg/m² range (45.37%), and the majority were multiparous (68.52%). According to S-POPQ staging, Stage III prolapse was most common (41.67%), followed by Stage II (21.30%). In terms of urinary incontinence, urgency urinary incontinence (27.78%) was frequently reported.

**Table 1:** Characteristics of women (*n* = 108)

Variables	Statistics
Age (Years)	54.69 ± 14.79 (Range: 27–85)
**Body Mass Index (kg/m** ^**2**^ **)**	
< 20	1 (0.93%)
20–25	38(35.19%)
25.1–29.9	49(45.37%)
≥ 30	20(14.81%)
**Parity**	
Nulliparous	6(5.56%)
Primiparous	12(11.11%)
Multiparous	74(68.52%)
Grand Multiparous	16(14.81%)
**S-POPQ Finding**	
Stage 0	14(12.96%)
Stage I	19(17.59%)
Stage II	23(21.30%)
Stage III	45(41.67%)
Stage IV	7(6.48%)
**Urinary incontinence**	
None	41(37.96%)
Stress UI	24(22.22%)
Urgency UI	30(27.78%)
Mixed UI	13(12.04%)
**Fecal Incontinence**	
None	103(95.37%)
Flatus	3(2.78%)
Fecal and Flatus	2(1.85%)
**Cough Test**	
Positive	14(12.96%)
Negative	94(87.04%)
**Pelvic floor disorders**	
With PFD	97(89.81%)
Without PFD	11(10.19%)

Table 2 summarizes baseline PFIQ-7 scores among 108 participants, showing significantly higher total and subscale scores in women with pelvic floor disorders (PFD) compared to those without.

**Table 2:** PFIQ-7 summary score at baseline

PFIQ-7	Statistics	Overall *n* = 108	With PFD *n* = 97	Without PFD *n* = 11
PFIQ.7 (Total)	**Mean ± SD**	**53.83 ± 49.27**	**59.45 ± 48.86**	**4.23 ± 6.03**
	**Median (IQR)**	**45.24(76)**	**47.62(71)**	**0**
**Subscale of PFIQ-7**				
Urinary Impact Questionnaire (UIQ-7)	Mean ± SD	24.15 ± 30.36	26.61 ± 31.07	2.51 **±** 5.58
	Median (IQR)	13.78(38)	19.05(45)	0
Colorectal-Anal Impact questionnaire (CRAIQ-7)	Mean ± SD	2.82 ± 12.30	3.14 ± 12.94	0
	Median (IQR)	0(0)	0(0)	0
Pelvic Organ Prolapse Impact Questionnaire (POPIQ-7)	Mean ± SD	26.68 ± 29.11	29.70 ± 29.23	1.73 ± 3.84
	Median (IQR)	19.05(46)	19.05(52)	0

PFIQ-7: Pelvic Floor Impact Questionnaire-7

### Evaluation of measurement properties

#### Content validity/face validity

In pilot testing, ten participants completed the questionnaire, assessing its length, clarity, cultural relevance, language, and ease of understanding. All questions displayed face validity, with participants finding them clear, comprehensive, and easy to understand. Each participant completed the questionnaire independently, recognizing the importance of the questions for clinical use in Pakistan. There were no missing responses, supporting near-excellent content and face validity.

#### Content Validity Index (CVI)

A multidisciplinary expert committee (6 members) assessment determined content validity. Following translation, the CVI was calculated using Waltz and Bausell’s [29] CVI approach. Expert consensus led to the development of the questionnaire’s Urdu version. The CVI value of 1 indicated excellent content validity.

### Feasibility

In terms of feasibility, all participants completed the Urdu version of the PFIQ-7 without any missing responses. Interviewers encountered no difficulties in administering the questionnaire, and participants reported no challenges in comprehending the items. The average duration required for the completion of the PFIQ-7 was approximately four to five minutes.

### Internal consistency

Cronbach’s alpha coefficient for the overall score of the Urdu version of the PFIQ-7 was 0.884, indicating good internal consistency. Additionally, the subscales had excellent internal consistency, with coefficients ranging from 0.908 to 0.985 (*p* < 0.0005). (Table 3).

### Test-retest reliability

Test-retest reliability was evaluated using the ICC. A subgroup of 40 women was reassessed two weeks after the initial administration. The total PFIQ-7 score exhibited an ICC of 0.995, while the subscale scores ranged from 0.992 to 0.999 (*p* < 0.0005), indicating excellent reliability (Table 3).

**Table 3:** Internal consistency and test-retest reliability of the PFIQ-7 questionnaires

PFIQ-7 questionnaires	Test–retest reliability (*n* = 40)		Internal consistency (*n* = 108)
	ICC	*P*-Value	Cronbach’s Alpha
**PFIQ-7**	0.995	0.0005	0.884
**Subscale of PFIQ-7**			
Urinary Impact Questionnaire (UIQ-7)	0.994	0.0005	0.939
Colorectal-Anal Impact Questionnaire (CRAIQ-7)	0.999	0.0005	0.985
Pelvic Organ Prolapse Impact Questionnaire (POPIQ-7)	0.992	0.0005	0.908

ICC: intra-class correlation

### Construct validity: known groups

By comparing PFIQ-7 scores, including those from its subscales (UIQ-7, CRAIQ-7, and POPIQ-7), construct validity was demonstrated. The total and subscale scores were considerably higher for women experiencing POP at both time points than women without POP (Mann–Whitney U Test; *p* < 0.0005). In a similar way, UI patients scored higher overall and on subscales compared to women without UI (Table 4).

**Table 4:** Comparison of total and subscale PFIQ-7 scores between women

Total and subscales of PFIQ-7	POP			Urinary incontinence		
	Yes *n* = 94	No *n* = 14	*P*-Value	Yes *n* = 67	No *n* = 41	*P*-Value
**PFIQ.7 (Total)**	61.14 ± 48.67	4.69 ± 6.88	0.0005	69.01 ± 52.43	29.01 ± 30.66	0.0005
**Subscale**						
UIQ-7	27.25 ± 31.29	3.33 ± 6.72	0.003	38.10 ± 3.79	1.37 ± 4.35	0.0005
CRAIQ-7	3.24 ± 13.14	0	0.229	3.06 ± 10.54	2.44 ± 14.87	0.315
POPIQ-7	30.65 ± 29.19	1.36 ± 3.45	0.0005	27.86 ± 29.13	25.20 ± 29.01	0.640

Results are presented as mean ± SD. Mann-Whitney U test applied

PFIQ-7: Pelvic Floor Impact Questionnaire-7, UIQ: Urinary Impact Questionnaire, CRAIQ: Colorectal–Anal Impact Questionnaire, POPIQ: Pelvic Organ Prolapse Impact Questionnaire

### Criterion validity

The analysis of Spearman’s correlation coefficients (SCC) demonstrated that both the total and subscale scores of the PFIQ-7 were significantly associated with S-POPQ vaginal examination findings (SCC test; *p* = 0.005). The S-POPQ and PFIQ-7 scores showed a strong correlation (SCC = 0.67; *p* = 0.005). Higher SCC values were specifically linked to the prolapse dimensions within the PFIQ-7, with the POPIQ subscale showing the strongest correlation (SCC = 0.682).

All predefined hypotheses were validated, supporting criterion validity. Women who had POP declared significantly higher POPIQ-7 scores (30.65 ± 29.19) compared to those without prolapse (1.36 ± 3.45) (Table 4). Furthermore, PFIQ-7 scores were shown to correlate strongly with S-POPQ findings, confirming criterion validity.

## Discussion

Our study has effectively translated and culturally adapted the PFIQ-7 into Urdu and evaluated its psychometric properties. The findings demonstrated near-excellent content and face validity, good internal consistency, strong test-retest reliability, significant construct validity, and very good criterion validity. All hypotheses regarding the reliability and validity of the Urdu version were confirmed, supporting its use as a standard instrument in clinical and research settings for women who speak Urdu and are experiencing PFDs.

The ease of responding to most items and the ability to complete the questionnaire within a brief period indicate its acceptability among respondents. The average completion time for the Urdu PFIQ-7 was approximately four to five minutes, while the completion times reported for the Amharic version were seven minutes [26], the French and Spanish versions reported completion times of 3.4 and 7.5 min, respectively [14, 30].

Our study also confirmed the high reliability of the Urdu PFIQ-7, with Cronbach’s alpha values for all subscales exceeding 0.90, indicating excellent internal consistency. These findings are consistent with other validated versions of the PFIQ-7. For example, similar Cronbach’s alpha values were reported for the Amharic [26], Tigrigna [23] Greek [22], Polish [31], Estonian [32], Sinhala [33], and Finnish [24] versions. While slightly lower but acceptable values were observed in the Turkish version (0.73–0.80) [17] and the Chinese version (0.64–0.70) [16], these results still support the reliability of the instrument across different cultural and linguistic adaptations. The two-week test-retest reliability analysis of the Urdu PFIQ-7 demonstrated exceptional consistency, with an ICC of 0.995 for the overall summary score (*p* < 0.0005). These results surpass those reported for the Amharic version [26], which showed an ICC of 0.86 (*p* < 0.001). Similarly, our findings were higher than those observed in other validation studies, including the Tigrigna version (ICC = 0.916) [23], Greek version (ICC = 0.840) [22], Finnish version (ICC = 0.75, range 0.62–0.84) [24], and Norwegian version (ICC = 0.83).^(34)^ The high test-retest reliability in this study confirms the consistency of the Urdu PFIQ-7 over time, reinforcing its reliability for repeated use in clinical and research settings.

The correlation between PFIQ-7 scores and S-POPQ findings was utilized to assess criterion validity, which was demonstrated to be strong (SCC = 0.67; *p* = 0.005). These results align with findings from the previous study [26], although they used different relative standards as a criterion. The study demonstrated a noteworthy association between objective vaginal examination results and PFIQ-7 scores. This observation is consistent with findings from other validation studies [20, 21, 34]. Notably, PFIQ-7 scores increased with higher POP-Q stages. However, the total PFIQ-7 scores in our study were comparatively lower than those found in prior research (Table 2). This may be attributed to the study population, which primarily comprised urban, educated women who likely had earlier access to healthcare services, greater awareness, and timely intervention for prolapse symptoms. This contrasts with studies involving rural or less-educated populations, where limited healthcare access often results in more severe symptoms and higher reported scores.

One of the strengths of this study is the substantial number of responses obtained for all the questionnaires administered. The broad age range of participants (27–85 years) further highlights the utility of the validated tools across different age groups. Additionally, including asymptomatic women in the study allowed for meaningful comparisons, with notable differences observed in the total and subscale scores of both instruments. These findings reinforce the validity and applicability of the PFIQ-7 for diverse populations.

The study does have certain limitations. The single-center design and small sample size are notable constraints. Nevertheless, the university hospital’s referral status and the lack of language and cultural diversity within the Urdu-speaking population mitigate some of these limitations. Still, conducting the study in an urban tertiary care hospital may restrict how broadly the results can be applied to rural or remote communities. Another limitation of our study is that we did not perform confirmatory factor analysis (CFA) to assess the structural validity of the translated PFIQ-7. While the original instrument has been rigorously validated with a well-established factor structure, including across various cultural settings, CFA remains an important step in cross-cultural adaptation studies. However, due to sample size constraints and the primary focus on assessing reliability and known-groups validity, CFA was not feasible in the current study. Future studies with larger and more diverse sample sizes, including participants from rural areas, are recommended to conduct confirmatory factor analysis (CFA) to validate the factor structure and confirm the broader applicability of the findings in this population.

## Conclusion

The research conducted establishes the validity and reliability of the Urdu version of the PFIQ-7 as an effective instrument for evaluating how POP affects women’s quality of life. The results complement the body of research on PFDs and offer valuable information for medical professionals involved in the management of POP in Pakistan. The ease of administration and practical utility of this questionnaire make it suitable for use in both clinical practice and research settings.

## Data Availability

The datasets used and/or analysed during the current study are available from the corresponding author on reasonable request.

## Abbreviations

PFIQ-7: Pelvic Floor Impact Questionnaire
PFDs: Pelvic floor disorders
PROMs: Patient-reported outcome measures
UI: Urinary incontinence
POP: Pelvic organ prolapse
FI: Fecal incontinence
HRQoL: Health-related quality of life
UIQ: Urinary Impact Questionnaire
CRAIQ: Colo-Rectal-Anal Impact Questionnaire
POPIQ: Pelvic Organ Prolapse Impact Questionnaire
COSMIN: Consensus-based Standards for the Selection of Health Measurement Instruments
S-POPQ: Simplified Pelvic Organ Prolapse Quantification
ICC: Intraclass correlation coefficients
SCC: Spearman’s correlation coefficient
CVI: Content Validity Index
